# A high throughput method for Monitoring of Sorafenib, regorafenib, cabozantinib and their metabolites with UPLC-MS/MS in rat plasma

**DOI:** 10.3389/fphar.2022.955263

**Published:** 2022-09-08

**Authors:** Er-Min Gu, Ya-Nan Liu, Lvjun Pan, Yingying Hu, Xuemei Ye, Pingping Luo

**Affiliations:** ^1^ The First People’s Hospital of Jiashan, Jiaxing, Zhejiang, China; ^2^ The First Affiliated Hospital of Wenzhou Medical University, Wenzhou, Zhejiang, China; ^3^ The People’s Hospital of Lishui, Lishui, Zhejiang, China

**Keywords:** hepatocellular carcinoma, tyrosine kinase inhibitors, drug metabolites, UPLC-MS/MS, pharmacokinetics in rats

## Abstract

As multi-targeted tyrosine kinase inhibitors, sorafenib, regorafenib and cabozantinib are widely used in hepatocellular carcinoma (HCC) for systemic therapies with anti-proliferative and anti-angiogenic effects. Nevertheless, adverse effects or insufficient efficacy appear frequently due to the plasma concentration with individual variability of these drugs. To ensure the curative effect and safety by therapeutic drug monitoring (TDM), this study developed a high throughput method to quantify sorafenib, regorafenib, cabozantinib and their active metabolites in plasma simultaneously. The chromatographic separation analysis achievement was performed on a Waters-ACQUITY UPLC BEH C18 column by UPLC-MS/MS system using a gradient elution of solvent A (acetonitrile) and solvent B (water with 0.1% formic acid) in 3.0 min. This method presented satisfactory results of specificity, precision (the intra-day coefficient of variation was between 2.5% and 6.6%, and the inter-day coefficient of variation was between 4.0% and 11.1%) and accuracy (within ±15% for intra-day and inter-day), as well as the stability under certain conditions, the matrix effect in plasma, and extraction recovery (75.6%–94.4%). The linearity of each analyte in the proper concentration scope indicated excellent. This study strictly complied with the performance rules of assay validation in biological medium proposed by FDA and was successfully applied to the pharmacokinetic study in rats. Thus, it would be an advantageous option to research the relationship between concentration-efficacy and concentration-toxic in HCC patients who were supposed to take these medications.

## Introduction

Hepatocellular carcinoma (HCC) is one of the most common malignancies (75%–85% of primary liver cancer) (Bruix et al., 2015; Villanueva, 2019) and is the third leading cancer related to death worldwide, which caused approximately 830,000 deaths and 906,000 new cases rose in 2020. HCC often results from cirrhosis and is closely related to chronic liver diseases that related to the risk factors like chronic infection by hepatitis B virus or C virus, obesity and heavy alcohol intake (Jors et al., 2015; Ma et al., 2021; Sung et al., 2021). Liver transplantation and surgical resection can be applied to only 10%–20% of HCC patients and mainly cure early liver cancer (Lin et al., 2012); but for advanced HCC patients who are unfit for curative treatment or local therapy, systemic therapies remain the primary treatment (Razumilava and Gores, 2014; Ferrante et al., 2020). The metastasis of cancer cells from the primary site to the tissues surrounded and even the organs distant is the dominant reason of related death (Lambert et al., 2017). At present, the most frequent chemotherapy resistance and adverse drug reaction become therapeutic challenges when at the advanced stage and result in poor prognosis.

Sorafenib as an oral tyrosine kinase inhibitor (TKI) for the first-line management to the patients with unresectable or advanced HCC has been approved since 2007 by FDA (Bangaru et al., 2020). However, most HCC patients during sorafenib treatment experience disease progression and they approximately maintain eight months in the overall survival time. Fortunately, the improvement of cancer sequential treatments has been verified in recent years (Personeni et al., 2019; Vogel and Saborowski, 2020; Song et al., 2021). For patients who tolerated sorafenib, regorafenib became the predominant oral multiple TKI to anti-angiogenic tyrosine kinase and blocked the activity of VEGFR (−1, −2, and −3), PDGFR, RAF1, KIT, and RET kinases to suppress cell proliferation, apoptosis induction and the inhibition of angiogenesis (Bruix et al., 2017). It is worth noting that regorafenib possibly due to its broader spectrum of kinases (e.g.,VEGFR-1, TIE2, RET) is quite superior to sorafenib (Heo and Syed, 2018). In 2019, cabozantinib was approved for previous treatment of HCC patients with sorafenib as a sequential treatment in the United States (Abou-Alfa et al., 2018). In contrast to regorafenib and sorafenib, cabozantinib has unique targets in addition to the mesenchymal-epithelial transition factor (MET) receptor and the “anexelekto” (AXL) receptor tyrosine kinase to resist antiangiogenic, especially MET has predicted value involved in HCC progression by the MET/HGF pathway (Kelley et al., 2020; Schoffski et al., 2020). The most common adverse events were similar, hypertension, and kind of gastrointestinal and dermatological toxicity, observed with TKIs in HCC patients. The drug related events included hand–foot skin reaction, hypertension, fatigue, anorexia, diarrhea, and increased blood bilirubin; and a proportion of the patients, which accounted for about 10%, discontinued treatment due to regorafenib related adverse reactions in the RESORCE trial (Bruix et al., 2017). In the CELESTIAL trial, the occurrence rate of serious adverse events in patients receiving cabozantinib compared with that of placebo to treat HCC was 50% vs. 37% and the rate of discontinuation due to adverse events from cabozantinib or placebo was 16% (Abou-Alfa et al., 2018).

Sorafenib and regorafenib are primarily mediated by CYP3A4 undergoing oxidative metabolism in liver, as well as glucuronidation mediated by UGT1A9 (Ghassabian et al., 2012; Fu et al., 2018). Cabozantinib, a minor extent by CYP2C9, is metabolized mostly by CYP3A4 that stimulated by cytochrome b 5 activity and produced metabolite cabozantinib N-oxide (Indra et al., 2020). Their main circulating metabolites in plasma, shows potency similar to that of prototype drugs *in vitro*. The enzyme CYP3A4 with the characteristic of genetic polymorphisms showed wide variability in metabolic activities (up to 60-fold), and resulted in severe drug toxicity, unpredictable adverse events or therapeutic failure (Hu et al., 2017). Therapeutic drug monitoring (TDM) can guide adjustment of drugs to the appropriate concentration range and optimize the therapeutic effect to maximization and the drug toxicity to minimum (Herviou et al., 2016; Verheijen et al., 2017). To pharmacokinetic studies of TKIs in clinical TDM, ultra-performance liquid chromatography tandem mass spectrometry (UPLC-MS/MS) is a suitable method for its high sensitivity and efficiency of quantification (Wu et al., 2020; Ying et al., 2020). However, the simultaneous quantification of sorafenib, regorafenib, cabozantinib and their active metabolites in plasma has not been reported in the previous studies. Therefore, this study developed a high-throughput, selective and efficient UPLC-MS/MS method with convenient pretreatment to detect these TKIs and their active metabolites in rat plasma, and investigated the pharmacokinetics of these drugs in rats.

## Experimental

### Chemical materials

**FIGURE 1:**
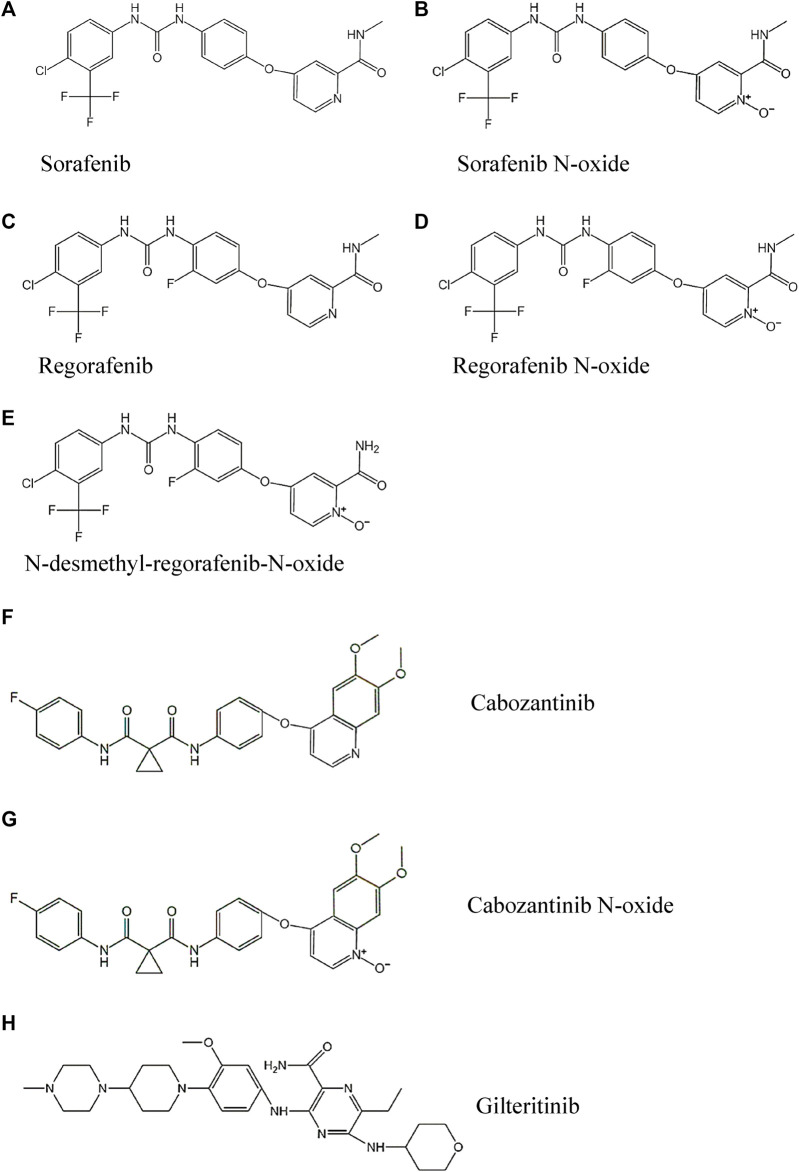
The chemical structures of the three TKIs, four active metabolites and IS studied: **(A)** Sorafenib, **(B)** Sorafenib N-oxide, **(C)** Regorafenib, **(D)** Regorafenib N-oxide, **(E)** N-desmethyl-regorafenib-N-oxide, **(F)** Cabozantinib, **(G)** Cabozantinib N-oxide and **(H)** Gilteritinib.

The standard analytes of sorafenib, regorafenib, cabozantinib, sorafenib N-oxide, regorafenib N-oxide, N-desmethyl-regorafenib-N-oxide, cabozantinib N-oxide (the purity of every analyte >98%) and gilteritinib (Internal standard, IS, purity >98%) were obtained from Shanghai Chuangsai Technology Co., LTD. (Shanghai, China), and their chemical structures were shown in Figure 1. Acetonitrile and methanol met LC grade were purchased from Merck Company (Darmstadt, Germany). Formic acid attained analytical grade was bought from Beijing sunflower and technology development CO., LTD. (Beijing, China). Distilled-water was dealt with A Milli-Q Reagent System (Millipore, Bedford, United States).

### Analytical conditions of UPLC-MS/MS

The Ultra Performance Liquid Chromatography system bought from Waters (Milford, MA, United States) was used for liquid chromatography including a binary solvent delivery manager I-CLASS, a sample manager FTN (kept at 10°C) and a column oven (maintained at 40°C). The crucial step of chromatographic separation was completed on a Waters-ACQUITY UPLC BEH C18 column (2.1 mm × 50 mm, 1.7 μm) attached a Waters ACQUITY UPLC BEH C18 VanGuardTM Pre-Column (2.1 × 5 mm, 1.7 μm). Mobile phase was consisted of acetonitrile (solvent A) and 0.1% formic acid in water (solvent B); flow rate was set at 0.40 ml/min and the procedure for gradient elution was adjusted as following: 10% solvent A at 0–0.5 min, 10%–90% solvent A at 0.5–1.0 min, and then maintained for 1.0 min for majorization, finally with 10% solvent A at 2.0–3.0 min for column equilibrating. The sample analysis time was 3.0 min entirely with 2.0 μl volume injection.

A triple quadrupole tandem mass spectrometer of Waters Xevo TQ-S (Milford, MA, United States) to quantificate the analytes by an electro-spray ionization (ESI) interface was adopted in positive ion mode. The selected multiple reaction monitoring (MRM) mode was operated to determine the transition, and the parameters collected from the mass spectrometric (MS) system with retention times of compounds were described in Table 1. The software of Masslynx 4.1 (Milford, MA, United States) matched with the UPLC-MS/MS system was used to conduct instrument control and achieve data acquirement.

**TABLE 1:** Specific mass spectrometric parameters and retention times (RTs) for the analytes and IS, including cone voltage (CV) and collision energy (CE).

Analytes	Precursor ion	Product ion	CV (V)	CE (eV)	RT (min)
Sorafenib	465.20	252.20	20	25	1.48
Sorafenib N-oxide	481.10	286.10	25	25	1.41
Regorafenib	483.30	270.20	20	21	1.49
Regorafenib N-oxide	499.10	304.10	20	26	1.42
N-desmethyl-regorafenib-N-oxide	485.20	202.10	20	26	1.39
Cabozantinib	502.20	323.10	30	36	1.27
Cabozantinib N-oxide	518.10	322.10	20	35	1.33
Gilteritinib	553.09	436.01	30	30	1.16

### Calibration curve and quality control

Prepared each analyte stock solution by dissolving the standards to 1.0 mg/ml in methanol separately and diluted the stock analytes with methanol to get concentration gradient of the quality control (QC) solutions and calibration curves. Meanwhile, IS working standard was obtained by diluting of stock solution with acetonitrile from 1.0 mg/ml to 50 ng/ml. Subsequently, a series of the calibration curve sample concentrations obtained by diluting the corresponding standard solutions with blank plasma to 100 μl (volume ratio 1:9), as 4–1,000 ng/ml for each analyte of sorafenib, regorafenib, cabozantinib and their active metabolites. Similarly, the QC samples were set at three concentrations (8, 80 and 800 ng/ml) for each analyte. All the stocks or working solutions were stored at 4°C and should be set at room temperature for 10 min at least before analyzing.

### Sample preparation

To prepare the samples, we used protein precipitation, a simple and mild method. In an Eppendorf tube in 2.0 ml size, 20 μl IS solution was added to 100 μl prepared plasma samples and the solution should be vortexed for 30 s. Then 300 μl acetonitrile was added to precipitate the plasma protein and the solution should be vortexed for 2.0 min, then followed with centrifugating 10 min at 13,000 × g in 4°C. For detection, 100 μl supernatant was transferred to the auto-sampler vials and just 2.0 μl sample applied to analysis in the UPLC-MS/MS system.

## Method verification

The bioanalytical assay got a reliable verification that complied with the light of the regulatory principles of the China Food and Drug Administration (CFDA), the guidelines of the United States Food and Drug Administration (US FDA), as well as the European Medicines Agency (EMA) (Xu et al., 2019; Tang et al., 2020).

The plots of calibration curve were depicted by the peak area ratios (y) of analytes to IS against the theoretical concentrations (x) and used a weight factor of 1/*x* ^2^ to suit the linear regression analysis. The lower limit of quantitation (LLOQ) of calibration curve to evaluate the sensitivity was investigated with signal to noise ratio (S/N)＞10 and required to achieve the criterion of accuracy and precision within 20%.

To determine the selectivity of this method, chromatograms of six blank close-relative rat plasma samples were analyzed to assess the endogenous interference on the analytes and IS within the retention times.

To investigate the effect of matrix effect, the peak areas of three respective analyte concentrations (8, 80, 800 ng/ml) in the treated blank plasma with corresponding standards dissolved in organic solvent was compared in the experiment. To estimate the extraction recovery, the comparison of the analyte with three concentrations (8, 80, 800 ng/ml) added to blank rat plasma before and after extraction was assessed. To determine the reliability of above extraction recovery and matrix effects, the above low, medium, high three different concentrations of QC experiments were repeated six times.

To evaluate the sample stability, four different possible conditions were tested: freeze/thaw completely three times, in the room temperature for 6 h, in the surrounding at 10°C for 12 h or −80°C for 28 days. The testing was determined by six duplicates of the seven QC analytes in three levels (8, 80, and 800 ng/ml) that dissolved in plasma.

### Pharmacokinetic application

Male Sprague-Dawley (SD) rats (weighted 200 ± 20 g) were purchased from the Animal Experiment Center of Wenzhou Medical University (Zhejiang, China). Before experiment, six rats were fasted for 12 h but water freely. This study of animal experiment was approved by the department of Animal Care and Use Committee of Wenzhou Medical University. Sorafenib, regorafenib and cabozantinib dissolved in 0.5% carboxymethyl cellulose sodium (CMC-Na), as a mixture dose, was orally administrated to rats with appropriate dose (40 mg/kg sorafenib, 16 mg/kg regorafenib and 6 mg/kg cabozantinib). Then approximately 300 μl blood samples, at times of 0.333, 0.667, 1, 1.5, 2, 3, 4, 6, 9, 12, 24, 36, 48, and 72 h orderly, were collected from rat tail vein and placed to tubes (contain heparin). Subsequently, the plasma was separated by centrifuging 10 min at 4,000 g in room temperature and then stored at 80°C for later analysis. The data of core pharmacokinetic parameters of the seven analytes in plasma was evaluated by Drug And Statistics (DAS) software version 3.0, bought from the Traditional Chinese Medicine of Shanghai University, in a non-compartmental model.

## Results and discussion

### Method development and optimization

In the present experiment, an analytical method that the seven analytes could be separated simultaneously by UPLC-MS/MS assay was validated through *in vivo* test with rat plasma. Mainly, the chromatographic conditions should be optimized to reduce the analysis time, improve the peak shape and the sensitivity. Firstly, in the process of selecting the organic phase, acetonitrile was chosen because it was advantageous in lower background noise and higher responses than methanol. Good peak and isolation shapes were obtained with 0.1% solution of formic acid (solvent B) and acetonitrile (solvent A) as the mobile phases. To improve the accuracy and reduce the experimental error, gilteritinib was selected as the IS. In terms of extraction technique, liquid–liquid extraction techniques or other traditional solid phase extractions were replaced and an operative protein precipitation approach was employed.

In order to remove protein and other potential interferences, a suitable pre-processing of samples was a critical step before UPLC-MS/MS analysis. Several solvents were tried for the present method, like trichloroacetic acid (concentration of 10%), perchloric acid (concentration of 6%), acetonitrile (concentration of 100%) or acetonitrile mixed with methanol. Finally, acetonitrile could provide optimized precipitation of protein extraction, more efficient and reproducible, for the analytes and IS from plasma.

### Selectivity, matrix effect and extraction recovery

**FIGURE 2:**
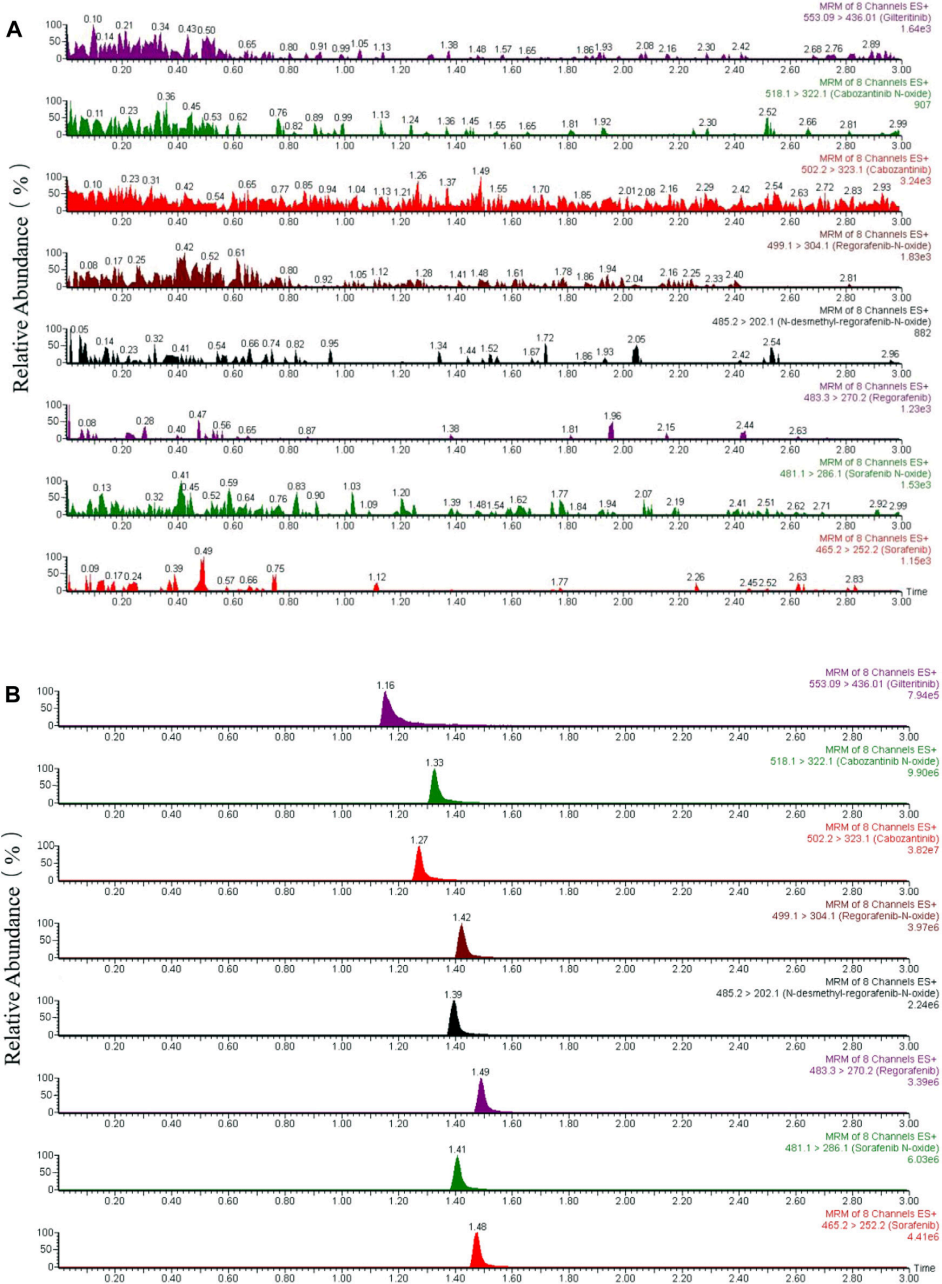
Representative chromatograms of sorafenib, regorafenib, cabozantinib, gilteritinib (IS) and metabolites of sorafenib N-oxide, regorafenib N-oxide, N-desmethyl-regorafenib-N-oxide, cabozantinib N-oxide in rat plasma. **(A)** One blank plasma; **(B)** blank plasma added standards of the seven analytes and IS.

The method for the selectivity confirmation had been shown on the typical chromatograms (Figure 2): the blank rat plasma sample (Figure 2A) and the blank plasma added standard analytes with IS (Figure 2B). As a result, the chromatograms exhibited the analytes at their respective time points, including the IS; and the interfering peaks of endogenous substances were not obvious and could be ignored. The findings of extraction recoveries and matrix effect, which achieved the average range of 75.6%–94.4% and 89.1%–114.2% as well as the relative standard deviation (RSD, %) for precision<15% and the relative error (RE, %) for accuracy within ±15%, were respectively shown in Table 2.

**TABLE 2:** Recovery and matrix effect of each analyte in SD rat plasma (*n* = 6).

Analytes	Concentration added (ng/ml)	Recovery (%)		Matrix effect (%)	
		Mean ± SD	RSD (%)	Mean ± SD	RSD (%)
Sorafenib	8	84.7 ± 11.1	13.1	104.0 ± 14.7	14.2
	80	91.9 ± 5.1	5.6	98.3 ± 6.9	7.0
	800	89.1 ± 4.4	5.0	91.9 ± 6.5	7.1
	8	79.9 ± 11.0	13.8	109.6 ± 14.1	12.9
Sorafenib N-oxide	80	86.7 ± 5.6	6.4	111.9 ± 11.1	10.0
	800	87.4 ± 4.3	5.0	107.2 ± 10.3	9.6
	8	76.0 ± 3.0	3.9	103.9 ± 13.9	13.3
Regorafenib	80	93.2 ± 6.6	7.1	92.4 ± 5.8	6.2
	800	94.4 ± 6.3	6.7	89.1 ± 7.9	8.9
	8	75.6 ± 4.2	5.6	114.0 ± 10.3	9.1
Regorafenib N-oxide	80	89.4 ± 6.0	6.7	113.8 ± 6.6	5.8
	800	89.8 ± 2.2	2.5	102.2 ± 10.0	9.8
	8	80.4 ± 9.0	11.2	112.9 ± 14.2	12.6
N-desmethyl-regorafenib-N-oxide	80	87.1 ± 4.4	5.1	109.4 ± 10.6	9.7
	800	88.0 ± 4.3	4.9	102.4 ± 9.6	9.4
	8	85.0 ± 8.5	10.0	114.2 ± 11.1	9.7
Cabozantinib	80	92.1 ± 4.0	4.3	112.3 ± 3.4	3.0
	800	92.7 ± 4.1	4.4	103.1 ± 12.9	12.5
	8	77.3 ± 8.2	10.5	110.5 ± 9.7	8.8
Cabozantinib N-oxide	80	86.2 ± 4.6	5.4	107.2 ± 14.1	13.2
	800	87.3 ± 4.4	5.1	111.2 ± 10.4	9.4

### Linearity and sensitivity

The test formulas of the calibration curves were established by the response ratio of analyte standards/IS (y) against the analytes in plasma (x). The linear correlation index of this method showed *r* ^2^ > 0.99 for each analyte and met the scope of detective concentrations on the calibration standard curve respectively. This method was quite content with the seven analytes for the quantification of LLOQ 4 ng/ml. The results of RSD% for precision within 20% and RE% for accuracy within ±20% were conformed to the standard requirements of error value.

### Accuracy and precision

Repeat six determinations with three gradients of QC samples (8, 80, and 800 ng/ml) were performed to assess the precision, and accuracy (shown in Table 3). In three days, the determinations of the accuracy and precision were all met the standard error value ranges (drifted within 15%).

**TABLE 3:** The determination of accuracy and precision of the analytes in rat plasma.

Analytes	Concentration added (ng/ml)	Intra-day		Inter-day	
		RSD (%)	RE (%)	RSD (%)	RE (%)
Sorafenib	8	6.6	9.6	9.4	12.0
	80	5.0	11.9	6.7	10.8
	800	3.3	−0.7	5.3	0.5
Sorafenib N-oxide	8	6.3	13.8	7.5	7.9
	80	4.3	12.2	5.1	7.7
	800	3.2	3.7	4.5	−1.1
Regorafenib	8	6.6	2.7	11.1	−5.6
	80	4.4	13.2	6.2	7.1
	800	3.3	3.6	5.0	−1.6
Regorafenib N-oxide	8	5.5	14.7	7.8	8.2
	80	3.8	14.7	5.0	10.7
	800	3.4	4.7	4.8	−0.1
N-desmethyl-regorafenib-N-oxide	8	4.6	11.0	6.0	7.3
	80	4.0	7.9	4.8	3.6
	800	3.7	2.8	4.1	−1.2
Cabozantinib	8	5.6	12.2	6.8	9.7
	80	4.4	12.8	4.9	9.3
	800	2.5	1.8	4.0	−1.9
Cabozantinib N-oxide	8	5.4	13.3	6.7	8.3
	80	3.7	9.4	4.7	5.5
	800	3.1	1.0	4.2	−3.3

### Stability

The stability of the stored extractions in four variable environments were tested: short period time at room temperature 25°C for 6 h, in the auto-sampler 10°C for 12 h, long time at −80°C for 28 days and three cycles of complete freeze/thaw. As a result, the stability displayed excellently well under the above four conditions (shown in Table 4).

**TABLE 4:** The stability of the analytes from plasma in rats under different conditions.

Analytes	Concentration added (ng/ml)	25°C, 6 h		10°C, 12 h		−80°C, 28 d		Freeze/thaw	
		RSD (%)	RE (%)	RSD (%)	RE (%)	RSD (%)	RE (%)	RSD (%)	RE (%)
Sorafenib	8	8.4	11.5	10.3	2.4	9.7	1.7	11.7	0.2
	80	7.3	14.0	5.5	−4.1	4.8	−3.8	5.3	−1.5
	800	4.6	−4.4	4.6	−12.1	2.4	−14.4	3.7	−11.7
	8	11.2	12.3	11.0	−0.2	10.3	−0.4	9.3	−2.6
Sorafenib N-oxide	80	4.3	1.6	3.8	−3.5	3.0	−3.8	2.7	−1.3
	800	3.1	−11.2	3.3	−13.3	1.2	−14.6	1.8	−12.4
	8	7.8	13.3	11.2	−14.3	11.8	−13.8	14.6	−3.7
Regorafenib	80	8.2	11.4	5.2	−8.1	3.9	−7.0	4.0	−5.6
	800	4.5	−4.6	3.7	−14.0	1.4	−16.3	2.8	−13.6
	8	12.9	15.0	12.2	0.4	8.2	−2.7	9.1	−3.7
Regorafenib N-oxide	80	6.2	5.6	5.2	−3.8	3.3	−3.6	3.2	−0.5
	800	2.9	−9.8	4.1	−12.8	2.4	−14.7	2.3	−12.2
	8	11.6	12.7	11.2	3.0	6.7	−1.4	10.7	−2.1
N-desmethyl- regorafenib-N-oxide	80	4.5	−1.2	6.9	−5.1	2.4	−5.9	3.2	−4.8
	800	3.0	−10.1	3.6	−12.0	0.7	−12.2	1.8	−10.5
	8	10.7	8.6	8.1	−0.6	3.9	1.2	3.3	−2.3
Cabozantinib	80	3.4	0.8	3.5	−3.2	2.8	−0.2	3.1	0.7
	800	2.1	−11.0	3.5	−11.9	0.8	−13.0	1.7	−11.0
	8	7.1	−1.0	9.7	−2.6	6.1	−3.1	5.4	−5.0
Cabozantinib N-oxide	80	4.0	−5.5	3.4	−7.1	3.0	−5.2	3.5	−3.4
	800	2.1	−13.7	3.3	−14.3	1.0	−14.9	1.6	−13.0

### Pharmacokinetics and pre-clinical relevance

**FIGURE 3:**
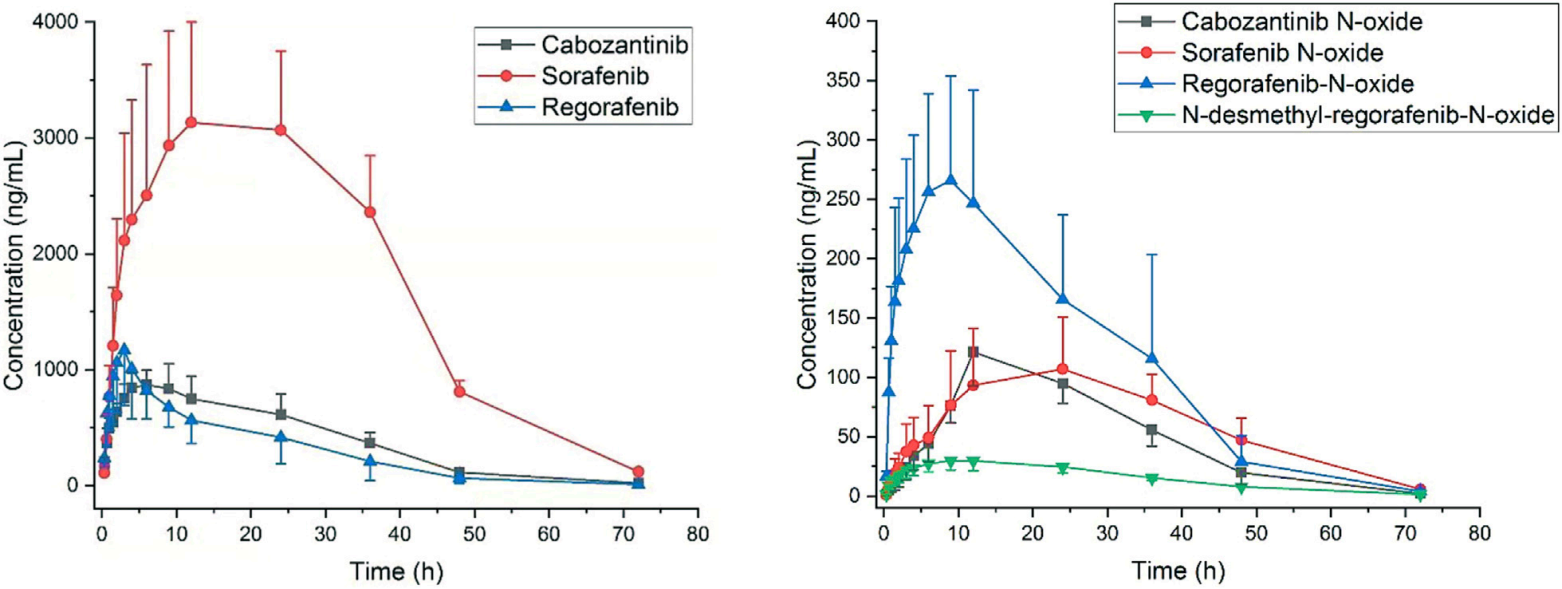
Plasma concentration to time after oral administration of sorafenib 40 mg/kg, regorafenib 16 mg/kg and cabozantinib 6 mg/kg in rats (Mean ± SD, *n* = 6).

This pharmacokinetic investigation was able to assess the plasma concentrations of three drugs and four of the corresponding active metabolites in rats. The plasma concentration-time curves after a single administration orally of sorafenib, regorafenib and cabozantinib were illustrated in Figure 3 and the capital pharmacokinetic parameters obtained from six rats, including the drug half-life (t_1/2_), peak time (T_max_), peak concentration (C_max_), plasma clearance (CL) and the other important parameter values were summarized in Table 5.

**TABLE 5:** Pharmacokinetic parameters of the prototype analytes and four metabolites in rats (Mean ± SD, *n* = 6).

Parameters	Sorafenib	Sorafenib N-oxide	Regorafenib	Regorafenib N-oxide	N-desmethyl-regorafenib-N-oxide	Cabozantinib	Cabozantinib N-oxide
t_1/2_ (h)	9.12 ± 1.36	11.97 ± 5.43	9.02 ± 1.20	8.52 ± 2.46	13.81 ± 3.29	10.26 ± 2.75	10.71 ± 2.56
T_max_ (h)	17.00 ± 7.98	24.00 ± 7.59	3.00 ± 0.01	12.75 ± 7.89	13.50 ± 7.14	7.17 ± 2.14	12.00 ± 0.01
C_max_ (μg/L)	3,426.48 ± 772.39	112.28 ± 44.53	1,170.07 ± 479.13	301.29 ± 66.08	31.62 ± 6.92	938.91 ± 92.33	121.36 ± 28.66
CL (L/h/kg)	0.32 ± 0.08	10.13 ± 4.69	0.83 ± 0.28	2.18 ± 0.89	14.09 ± 2.07	0.23 ± 0.06	1.70 ± 0.29
AUC_0→t_ (μg/L •h)	127,597.50 ± 25,558.18	4,363.44 ± 1,552.46	21,672.53 ± 9,362.04	8,093.29 ± 2,692.11	1,107.54 ± 182.07	27,542.81 ± 5,632.90	3,536.77 ± 580.95
AUC_0→∞_ (μg/L •h)	129,231.35 ± 25,503.07	4,623.17 ± 1820.30	21,812.85 ± 9,465.08	8,143.11 ± 2,695.34	1,154.83 ± 176.08	27,903.28 ± 5,670.75	3,616.77 ± 626.17
Vd (L/kg)	4.26 ± 1.29	160.67 ± 63.85	10.80 ± 4.05	28.20 ± 16.85	281.31 ± 80.12	3.29 ± 0.97	25.68 ± 4.98
MRT_0→t_ (h)	24.81 ± 1.77	28.73 ± 2.86	16.58 ± 2.35	19.18 ± 4.54	23.64 ± 1.45	20.48 ± 0.90	23.73 ± 1.33
MRT_0→∞_(h)	25.59 ± 1.93	29.57 ± 3.31	16.99 ± 2.36	19.63 ± 4.26	25.03 ± 1.45	21.34 ± 1.47	24.02 ± 1.28

For HCC patients, most adverse events due to the inter-individual significant variability of plasma concentration of these TKIs could be managed with dose modifications or temporary cessation treatment. These oral drugs present different bioavailability, with 69%–83% for regorafenib and 38%–49% for sorafenib, and are mainly metabolized by cytochromes P450 3A4 (CYP3A4) in human liver. CYP3A4, as a significant role in the oxidation of drugs, has the characteristic of individual difference with various subtypes of metabolic efficiency. On the other hand, these TKIs generate active metabolites sorafenib N-oxide, cabozantinib N-oxide and so on (Thillai et al., 2017), which can be accumulated notably in severe renal insufficiency or gastrointestinal disorder when treating for HCC patients. Moreover, these drugs may cause drug or food interactions (competition induced or CYP3A4 inhibited) and reduce the therapeutic effect or increase the occurrence of adverse reactions (Gillani et al., 2015; Ghassabian et al., 2019). UPLC-MS/MS is a preferred analytical method for the quantification of TKIs, because its high separation efficiency of chromatography and high accuracy and selectivity of the mass spectrum. To improve the efficacy of TKIs by TDM about the concentration-efficacy and concentration-toxic relationship in HCC patients, this study developed a specific UPLC–MS/MS method for the determination of sorafenib, regorafenib, cabozantinib and four of their active metabolites in rat plasma to support the pre-clinical exploration of drug concentration monitoring. Although sorafenib either alone or in combination with other TKIs has been monitored quantitatively *in vitro* and/or *in vivo* by UPLC-MS/MS (Allard et al., 2017; Li et al., 2021; Ye et al., 2021), there is still no method reported to analyze these seven materials simultaneously. In modern treatment options, regorafenib, or cabozantinib is often used for optional choices when insufficient efficacy appears after sorafenib treatment of HCC patients (Ueshima et al., 2017; Deeks, 2019). Since novel oral TKIs have become available, an analytical method had to be developed to analyse these drugs, preferably in a single run. Therefore, our laboratory had implemented a bioanalytical method for the measurement of sorafenib, regorafenib, cabozantinib and four of their active metabolites in a single run for pharmacokinetic evaluation and for individual patients.

## Conclusion

In summary, the developed and validated UPLC-MS/MS method has the characteristics of highly sensitive, specific, reproducible and high-throughput to quantitate sorafenib, regorafenib, cabozantinib and their active metabolites in simultaneous detection. The applicability of the UPLC-MS/MS analytical method has also been established to investigate the pharmacokinetics of these inhibitors of tyrosine kinase and their four active metabolites in rats. The method implemented a sample preparation by one-step protein precipitation with adequate recovery, and no significant matrix effect was observed. It provided superior sensitivity with LLOQ of 4 ng/ml for each analyte in the analysis and been successfully applied to PK studies in rats.

## Data Availability

The original contributions presented in the study are included in the article/supplementary material, further inquiries can be directed to the corresponding author.
